# AMPK and SNF1: Snuffing Out Stress

**DOI:** 10.1016/j.cmet.2007.10.001

**Published:** 2007-11-07

**Authors:** D. Grahame Hardie

**Affiliations:** 1 Division of Molecular Physiology, College of Life Sciences, University of Dundee, Dow Street, Dundee DD1 5EH, Scotland, UK

## Abstract

AMP-activated protein kinase (AMPK) has attracted much attention for its key role in energy homeostasis. Three new papers providing structural information on mammalian and yeast AMPK homologs give insights into the binding of the regulatory nucleotides AMP and ATP and how mutations are associated with cardiac glycogen storage disorders.

## Main Text

In mammals, metabolic stresses that inhibit ATP synthesis (e.g., hypoglycemia) or accelerate ATP consumption (e.g., muscle contraction) cause increases in the cellular AMP:ATP ratio that activate the AMP-activated protein kinase (AMPK) system. AMPK protects cells against such stresses by activating alternate catabolic pathways and inhibiting cell growth and division. This cellular energy-sensing role is most likely the function for which the AMPK system originally evolved. However, in multicellular organisms it also plays a key role in the regulation of whole-body energy balance, being involved in the control of food intake and energy expenditure by mediating effects of hormones like leptin and adiponectin (Kahn et al., 2005). Three new papers (Amodeo et al., 2007; Townley and Shapiro, 2007; Xiao et al., 2007) provide important structural information about AMPK and its yeast homologs.

AMPK exists as heterotrimeric complexes containing a catalytic α subunit and regulatory β and γ subunits. Binding of AMP to two domains on the γ subunit (Scott et al., 2004) activates the kinase both by direct allosteric activation and by inhibiting dephosphorylation of an activating site on the α subunit, effects that are antagonized by ATP (Hardie, 2007). Genes encoding α, β, and γ subunit orthologs are found in almost all eukaryotes. The role of the budding yeast *Saccharomyces cerevisiae* AMPK ortholog (the SNF1 complex) is known from genetic studies, being required for the response to glucose starvation, a stress that causes activation of the kinase and correlates with dramatic increases in cellular AMP:ATP (Wilson et al., 1996). Surprisingly, however, attempts to demonstrate activation of the yeast complex by AMP have so far failed.

**Figure 1:**
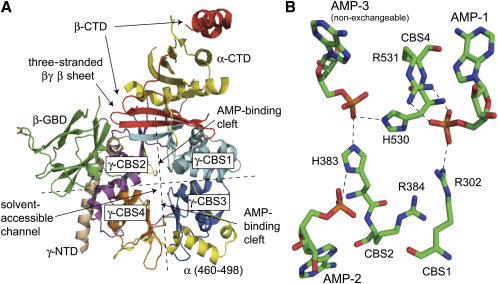
Structures of AMPK and Its Homologs (A) Cartoon view of the *S. cerevisiae* structure (Protein Data Bank ID code 2QLV) illustrating the general layout of the secondary structural elements. The dotted lines represent two of the three axes about which the cystathionine β-synthase (CBS) motifs of the γ subunits show approximate two-fold symmetry. The three-stranded β sheet connecting the β and γ subunits, the solvent-accessible channel through the γ subunit, and the clefts where AMP binds in mammalian AMPK between CBS1/CBS2 (N-terminal Bateman domain) and CBS3/CBS4 (C-terminal Bateman domain) are indicated. Key: α-CTD and α (460–498), Snf1 C-terminal domain and residues 460–498 (shown in yellow); β-CTD, Sip2 C-terminal domain (red); β-GBD, Sip2 glycogen-binding domain (green); γ-NTD, Snf4 N-terminal domain (tan); γ-CBS1/2/3/4, Snf4 CBS motifs 1/2/3/4 (1, light blue; 2, purple; 3, dark blue; 4, orange). (B) “Stick” view (C atoms shown in green; N, blue; P, orange; O, red) of AMP1 and AMP3 (in the CBS3:CBS4 cleft) and AMP2 (in the CBS1:CBS2 cleft) in the mammalian α1β2γ1 complex (PDB ID code 2V8Q). Basic residues (using γ2 numbering) that interact with the phosphate groups of AMP or ATP are also shown. Interestingly, H383 (in CBS2) interacts with AMP molecules bound to both Bateman domains, while R302 (in CBS1) interacts with AMP in the other Bateman domain. R384 does not interact with AMP but does interact with ATP when it replaces AMP2 (Xiao et al., 2007). Figures were generated using MacPyMOL.

The new reports provide structures for the “core” of the αβγ complex from mammals and budding yeast (Xiao et al., 2007; Amodeo et al., 2007) that were solved by molecular replacement using a recent structure from the distantly related fission yeast *Schizosaccharomyces pombe* (Townley and Shapiro, 2007). Unfortunately, the complete αβγ complexes failed to crystallize, and the mammalian and fission yeast structures, while containing the entire γ subunits, only have the C-terminal regions of the α and β subunits. The budding yeast structure also contains the β subunit glycogen-binding domain and a small additional region of α (Figure 1A), but all three structures lack the kinase domain. Despite the fact that neither of the yeast kinases appears to be activated by AMP, they were nevertheless crystallized in the presence of the nucleotide, and AMP was found to be present in the crystals.

The structures of the three core complexes are very similar. The C-terminal region of the α subunit forms a compact domain around which the C-terminal region of the β subunit is wrapped. The main contact between this αβ complex and the γ subunit is via an intersubunit β sheet, of which the C-terminal end of the β subunit provides two strands and the γ subunit the third. It had previously been proposed that each of the two AMP-binding domains in the γ subunit (termed Bateman domains) is formed by association of two sequence repeats known as cystathionine β-synthase (CBS) motifs, with the clefts between the repeats providing the nucleotide-binding sites (Scott et al., 2004). While this idea has now been confirmed, the new structures also show that the two Bateman domains associate together in a pseudosymmetrical head-to-head arrangement, forming a flattened disk with a small solvent-accessible channel through the center that would potentially allow access of nucleotides from either side (Figure 1A). The nucleotides bind with their phosphate groups in this central channel. An interesting feature of this arrangement is that residues in one Bateman domain contribute to binding of nucleotide in the other (Figure 1B).

Because the two CBS motifs in each Bateman domain also associate together in a pseudosymmetrical manner, there are two potential nucleotide-binding sites in each domain, making four in each γ subunit. AMP occupies three of these in the mammalian structure, one in fission yeast, and none in budding yeast (although low-resolution data on a second crystal suggested the presence of one AMP in the same position as in fission yeast). The results with the mammalian complex were surprising because it has previously been proposed that the γ subunits bind only two molecules of each nucleotide (Scott et al., 2004). However, new binding studies with the mammalian αβγ complex confirm that only two bind reversibly (Xiao et al., 2007). This suggests that the third AMP is tightly bound and nonexchangeable, an idea supported by two additional findings: (1) the mammalian complex expressed in bacteria already contained one mole of bound nucleotide per mole of protein, primarily AMP, and (2) when ATP was soaked into crystals containing AMP, it replaced AMP at only two of the three sites (Xiao et al., 2007). The remaining AMP was bound in the same position as the single molecule observed in the yeast structures. The fourth potential site may be unoccupied in the human γ subunit because aspartate residues whose side chains interact with the 2′ and 3′ hydroxyls of the AMP ribose in the other three sites (D89, D244, and D316) are replaced by an arginine (R170). These residues are highly conserved in all eukaryotes.

Point mutations of up to ten different residues in the γ2 isoform of mammalian AMPK cause heart disease characterized by excessive glycogen storage in cardiac myocytes, with the key clinical feature being ventricular pre-excitation (Akman et al., 2007; Arad et al., 2007). A transgenic mouse model has suggested that the elevated glycogen storage is due to increased basal activity of the mutant complex, leading to increased glucose uptake in the absence of increased energy demand (Luptak et al., 2007). The disease-causing mutations all reduce binding and activation by AMP (Scott et al., 2004), but the increased basal activity is likely due to failure to bind the inhibitory nucleotide, ATP (Burwinkel et al., 2005). Satisfyingly, nine of the ten residues affected by the disease lie very close to the AMP/ATP binding sites, and six (including four basic residues, R302, H383, R384, and R531, human γ2 numbering) make direct contact with the nucleotides (Figure 1B). These six residues are conserved in all known vertebrate and invertebrate γ subunits, but R302 and H383 are not conserved in the yeast sequences. Since these two residues are involved in binding the two exchangeable molecules of AMP (Figure 1B), this may explain why AMP does not activate the yeast complexes. However, this begs the question as to why the latter still have Bateman domains that bind a single molecule of AMP.

The budding yeast structure contains a small additional region of the α subunit that interacts with the γ subunit and has been proposed to play a regulatory role (Jiang and Carlson, 1996). However, the explanation as to how AMP activates the mammalian kinase, while ATP inhibits it, remains elusive. There is no major change in conformation between the complexes containing ATP versus AMP, although the nucleotide phosphate groups are partially exposed to solvent, thus altering the distribution of surface charge. Xiao et al. (2007) propose that this may cause changes in interactions with the α or β subunits. However, none of the new structures contains the kinase domain, so its crucial interactions with the other domains remain unclear. With a system so exquisitely regulated, it was perhaps inevitable that even three new structures (plus previous structures of the isolated kinase, glycogen-binding, and Bateman domains) would not provide all of the answers. Explaining how AMP regulates kinase activity and dephosphorylation remains an important challenge for the future.
